# Wi-Fi signal for soil moisture sensing

**DOI:** 10.1007/s10661-024-13587-x

**Published:** 2024-12-27

**Authors:** Adil K. Salman, Mohammed Al-Jumaili, Magdalena Sut-Lohmann, Wolfgang Durner

**Affiliations:** 1 Division of Soil Science, Institute of Geoecology, TU Braunschweig, Brunswick, Germany; 2 Institute of Robotics and Process Control, TU Braunschweig, Brunswick, Germany

**Keywords:** Soil moisture, Soil sensing, Water content, Wi-Fi

## Abstract

Measuring soil moisture is essential in various scientific and engineering disciplines. Over recent decades, numerous technologies have been employed for in situ monitoring of soil moisture. Currently, dielectric-based sensors are the most popular measurement technology and provide acceptable accuracy for various measurement purposes. However, these sensors are relatively expensive, and alternative technologies, which are cheaper, are not accurate enough for scientific purposes. Recently, the idea of using a Wi-Fi signal to measure soil moisture has been presented. Theoretically, the use of Wi-Fi technology in soil sensing follows the same concepts as the previous dielectric sensors. The main advantage of Wi-Fi technology is the possibility of providing a relatively accurate and cost-effective solution for soil moisture measurement. In this work, we try to investigate the possibility of using Wi-Fi signal characteristics for soil sensing. Therefore, a series of small-scale laboratory and field experiments were conducted to test the concept. The results of these experiments were promising, showing strong linear relationships between Wi-Fi signal properties (received signal strength indicator, RSSI) and soil water content, with *R*^2^ values ranging between 0.92 and 0.99, indicating a strong correlation. They also illustrate the possibility of using this technology to develop an inexpensive and accurate device for measuring soil moisture. However, observations from the experiments also point to problematic factors involving the hardware and software used in the measurements. It is important to control these factors in the next steps to develop a reliable measurement device.

## Introduction

Precisely determining soil moisture levels plays a crucial role in enhancing the management of irrigated agriculture and the meticulous illustration of water balance, whether across soil horizons or throughout expansive watersheds. This measurement holds significant importance across diverse scientific and engineering applications (Agyeman et al., 2023; Salman et al., 2021). The standard method used to estimate soil water content is the gravimetric method, which serves as a benchmark for calibrating other techniques (Johnson, 1992). This method quantifies the water mass extractable from soil samples dried at 105 °C relative to their dry mass. However, direct soil moisture measurement proves impractical for monitoring due to several drawbacks: (i) Results are slow to obtain, causing discrepancies as soil moisture levels change dynamically. (ii) This method is intrusive, disrupting soil integrity, which poses challenges for hi-resolution temporal monitoring or small-scale studies. (iii) Its implementation is costly, involving significant labor and equipment expenses. (iv) In situ soil moisture monitoring and smart irrigation controllers are unfeasible using this method (Salman et al., 2021). Thus, various technologies of automated probes and sensor systems for monitoring soil moisture exist (Jackisch et al., 2020). Over recent decades, advancements in soil moisture sensing technologies have led to the development of indirect measurement methods. These include neutron moderation, nuclear magnetic resonance (NMR), electric resistance, and dielectric sensors (Hernández et al., 2019).

### Radio frequencies (RF) for soil sensing

Currently, dielectric sensors stand out as the most popular sensor principle in use today, focusing on assessing soil apparent permittivity (dielectric constant) to estimate soil water content (Topp, 2003). Additionally, these sensors are also utilized to measure the soil’s apparent electrical conductivity (EC).

The relative permittivity of specific material ${\varepsilon }_{r}$ described by David and Moses (2011) as

1 $${\varepsilon }_{r}=\varepsilon /{\varepsilon }_{0}$$

where $\varepsilon$ is the dielectric permittivity of the material and ${\varepsilon }_{0}$ is the permittivity of free space, with $\varepsilon_0=8.854\:\times\:10^{12}F/m$.

Sharma (2018) reported the successful application of various technologies for estimating soil permittivity to sense soil moisture, such as frequency domain reflectometry (FDR), time domain reflectometry (TDR), amplitude domain reflectometry (ADR), time domain transmission (TDT), and capacitance sensors. All these systems utilize the properties of RF signals to estimate soil characteristics (Salman et al., 2021).

In soil, RF propagation is characterized by slower speed and attenuation compared to air, primarily influenced by variations in permittivity (Huang et al., 2020). The relation between RF propagation velocity $v$ and permittivity ${\varepsilon }_{r}$ explained by Thomas (2010) as

2 $${v}_{soil} =\frac{{v}_{air}}{\sqrt{{\varepsilon }_{r}}}$$

where $v$ indicates velocity and ${v}_{air}=$ 3 × 10^8^ m/s.

It has been reported (Souto et al., 2008) that at 20 °C, the permittivity values ${\varepsilon }_{r}$ are approximately 1 for air, 3–7 for solid soil material, and 80 for water. This indicates that RF velocity slows down by ninefold in water and 2.5 times in dry soil material compared to air. This suggests that RF velocity in soil is closely tied to water content (Salman et al., 2021). It is worth noting that the EC of soil solution, which depends on the ionic content—including soluble salts and other ions—as well as temperature, also influences soil permittivity. Soil, characterized as a porous medium, comprises three phases: solid, liquid, and gas, each with its own dielectric constant (Cihlar and Ulaby., 1974). A three-phase integrating model can effectively illustrate how these soil components (air, solids, and water) collectively contribute to the bulk soil’s dielectric constant.

3 $${{\varepsilon }_{r}}^{\alpha }={{\varepsilon }_{a}}^{\alpha }{f}_{a}+{{\varepsilon }_{s}}^{\alpha }{f}_{s}+{{\varepsilon }_{w}}^{\alpha }\theta$$

The relative permittivities ${\varepsilon }_{a}$, ${\varepsilon }_{s}$, and ${\varepsilon }_{w}$ correspond to air, soil particles, and water. ${f}_{a}$, ${f}_{s}$, and $\theta$ denote their volume fractions (where *θ* is the volumetric water content in soil), and $\alpha$ ranges between 0 and 1 (Gardner et al., 1998). An empirical equation has been presented by Topp et al. (1980) to link ${\varepsilon }_{r}$ to the volumetric water content $\theta$ in soil:

4 $$\theta =-5.3\bullet {10}^{-2}+2.92\bullet {10}^{-2}{\varepsilon }_{r}-5.5\bullet {10}^{-4}{{\varepsilon }_{r}}^{2}+4.3\bullet {10}^{-6}{{\varepsilon }_{r}}^{3}$$

The permittivity of water is also a function of temperature. Cihlar and Ulaby (1974) define the relationship as

5 $$\varepsilon_w=88.6-0.368T$$

where $T$ is the temperature in °C.

RF signal attenuation through its path in soil is influenced by the soil attenuation coefficient and the travel distance. Bogena et al. (2009) suggested a linear model to describe RF signal attenuation in soil:

6 $${A}_{tot}=\beta d+{R}_{c}$$

where ${A}_{tot}$ is the total signal attenuation (dB), $\beta$ is the soil attenuation coefficient (dB/m), $d$ is the travel distance (m), and ${R}_{c}$ is the signal attenuation due to the reflectance by soil.

A higher EC in soil solution results in additional transmission losses in the RF signal, potentially causing an overestimation of soil moisture. This effect is particularly noted in capacitance sensors (Baumhardt et al., 2000; Salman et al., 2021; Schwank & Green, 2007; Thompson et al., 2007). Gardner et al. (2001) stated that the influence of EC on soil moisture measurement is expected for the sensors that operate with frequencies < 50 MHz and the effects of EC are negligible at higher frequencies. However, Salman et al. (2021) and Thompson et al. (2007) reported a significant influence of EC on the measurements of volumetric water content (VWC) by capacitance sensors operating at 80 and 100 MHz, respectively.

### Introducing Wi-Fi signal for soil sensing

Wi-Fi is a trademarked term defined by Song and Issac (2014) as a short-range wireless transmission technology that uses wireless methods to interconnect personal computers, hand-held devices, and other terminals. TechTerms (https://techterms.com/definition/wi-fi) defines Wi-Fi as “a wireless networking technology that allows computers and other devices to communicate over a wireless signal. It describes network components that are based on one of the 802.11 standards developed by the IEEE and adopted by the Wi-Fi Alliance.” This technology is extensively used today to connect and share information among various devices in our daily lives, including laptops, desktops, tablets, smartphones, smartwatches, car keys, printers, smart TVs, drones, video cameras, and more. It facilitates information exchange between these devices, forming a network known as the Internet of Things (IoT).

In October 2019, Microsoft researchers introduced the possibility of using Wi-Fi signal properties as an indicator to sense soil moisture and bulk electrical conductivity (Ding & Chandra, 2019a, 2019b). The measurement technology follows the same basic concepts (signals slow down and are attenuated when passing through soil in response to changes in soil permittivity, which are influenced by water content) as previous RF sensors. However, currently available sensors are all related to signal propagation along waveguides and thus sense soil moisture in the close vicinity of the sensors. The suggested idea (Ding & Chandra, 2019a, 2019b) provides an alternative in that it senses the wave propagation through free soil. Furthermore, it has the promise of being a cheap solution for sensing soil moisture because the cost of Wi-Fi transmitters and receivers is very low in comparison to the previous technologies. The suggested scheme can be described briefly by burying three Wi-Fi receivers at different depths under the soil surface to receive a signal transmitted from a transmitter (which can be a smartphone) located in the air over the soil surface. The time of fly (relative time of fly) between the transmitter and the receivers is estimated and used as input to calculate signal speed (known flying distances and fixed transmitting angle) that can be used to estimate the soil permittivity, which is finally used to calculate soil moisture and bulk electrical conductivity.

The idea looks promising with respect to decreasing the cost of soil monitoring devices (accuracy and precision will be an addition). Even more promising is the hope to eventually come up with a measuring technique that is no longer dependent on the perfect embedding of the sensors in a representative soil unit, which might be the key problem for getting reliable absolute water content from permittivity measurements (Durner, 2023).

### Concept to use the Wi-Fi signal for soil sensing

Our key idea is to simplify the principle of Ding and Chandra (2019a) further by just using the attenuation of a signal as a measure for moisture content. Of course, the attenuation will depend on many factors, some of which are related to the signaling and others to water-content-independent soil properties. Crucially, the confounding factors related to the measurement technique as such (signal generation technique, signal propagation to the antenna, type and orientation of the transmitting and receiving antenna, to name but a few) can in practice be kept constant and thus eliminated by a strictly defined measurement setup. One of the main advantages of the proposed concept is the flexibility in terms of the volume of the soil sample estimated with this technology compared to existing dielectric sensors. The size of the soil sample in our concept depends on the distance between the transmitter and receiver, which can vary from a few centimeters to a meter or even more with additional setup. This is a very important aspect in terms of the representativeness of the sensed soil volume. Compared to the existing TDR, FDR, and capacitance sensors, it has an advantage. The existing sensors estimate the moisture content of the soil within a radius of about 10 mm around the rods (TDR) or 20 mm (FDR and capacitance sensors) (Evett et al., 2008). However, the sensitivity zone is sometimes larger, depending on the probe design, such as in the case of antennas.

The most common Wi-Fi signal bands today are 2.4 (2.4–2.4835 GHz) and 5.8 (5.15–5.825 GHz). The 5.8 has the fastest data transmission speed, the lowest noise, and the highest number of non-overlapping channels. The 2.4 GHz frequency band has a longer range and is more effective at penetrating solid objects (Desa et al., 2009; Talvitie et al., 2015). Accordingly, the 2.4 signal is expected to travel a longer distance through the ground. On the other hand, signal absorption by water is higher at a frequency of 2.4 GHz.

To prove the concept, we have started some basic experiments with commonly available components for sending, receiving, and analyzing Wi-Fi signals.

## Materials and methods

The monitoring of soil moisture under field conditions constitutes the ultimate objective of the proposed approach. In pursuit of this objective, a sequence of five experiment types, increasing in complexity regarding structure and materials, was conducted. The first experiment took place in the laboratory using pure quartz sand, a material containing few problematic constituents. The second experiment, also conducted in the laboratory, utilized natural soil. In a third experiment in the lab, the influence of salt in the material was explicitly investigated. Finally, two field experiments were conducted. The first assessed two key aspects: the extent of signal propagation through natural soil and the correlation between signal attenuation and the length of the signal path within the soil. The second experiment aimed to determine the integrated soil water content from the soil surface to a target depth. The experiments are presented below.

### Quartz sand experiment

A correlation between Wi-Fi signal strength and volumetric water content in quartz sand media was established in a small pot experiment. The quartz sand was thoroughly washed with distilled water, dried in an oven, and then filled into a small plastic pot with a dry bulk density of 1.6 g/cm^3^. The quartz sand was chosen for the first experiment to avoid interaction with salts and organic components in the natural soil, which are known to have an influence on permittivity. This is one of the reasons for the widespread use of quartz sand in the factory calibration of sensors. Table 1 describes the materials and equipment used.

**Table 1:** Materials/devices used in quartz sand experiment

Materials/devices	Specification/roles
Quartz sand	Pure white quartz sand, 0.1–0.3 mm particle diameter, no organic content
logilink 5/2.4G dual-band Wi-Fi adapter	Installed inside the sand to be the source of the transmitted signal. [Conrad electronics, < 10 €]
USB cable	1.6 m USB extension cable to connect the adapter to the desktop computer
Desktop computer	Connected to the Wi-Fi adapter through a USB cable to control the on/off switch of the Wi-Fi signal
Laptop computer	Used as Wi-Fi receiver to measure the signal strength using “Wi-Fi analyzer software”
Sample containment	A plastic pot with a cylindrical shape, measuring 120 mm in diameter and 120 mm in height, was used to contain the sample

The Wi-Fi adapter (transmitter) was inserted into a plastic tube and sealed with a waterproof adhesive to prevent water from entering the tube. The tube was then placed in the plastic pot so that it was in the center of the quartz sand sample. We note that the sample size used in this experiment, with dimensions of 120 mm in diameter and 120 mm in height (Table 1), as well as the multi-environment signal path, may influence the accuracy of the measurements. However, we recognize that this laboratory setup serves as an indicator and that natural conditions in the field can differ significantly from the controlled laboratory environment.

**Fig. 1:**
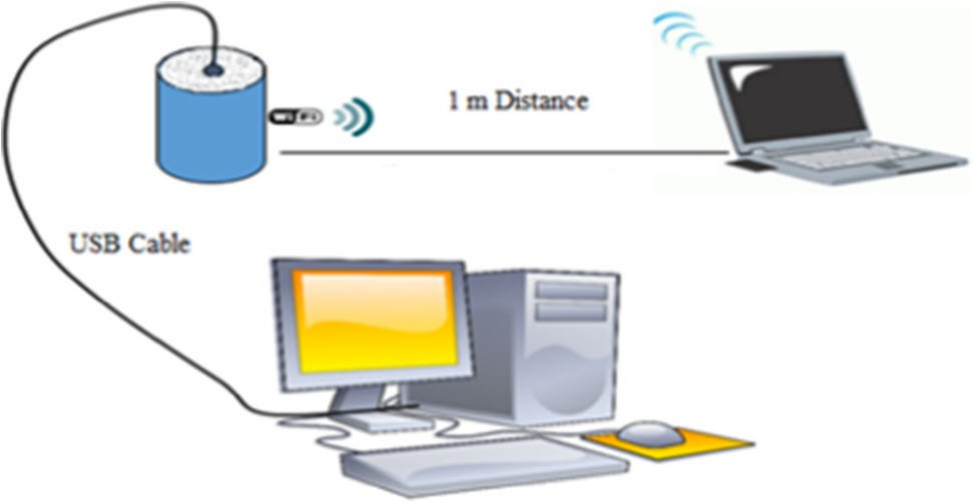
The experimental setup of the quartz sand experiment

The transmitter was connected to a desktop computer via a USB cable (Fig. 1). The sample was saturated with distilled water and then left to evaporate naturally. A balance was used for daily monitoring of changes in mean moisture content. Every day, directly before weighing, the received signal strength indicator (RSSI) was assessed (measured by decibel-milliwatts, dBm) with a laptop at a horizontal distance of 1 m from the pot.

The computer was equipped with appropriate software for measuring the RSSI, specifically, a Wi-Fi analyzer developed and published by Matt Hafner (https://matthafner.com/wifi-analyzer). The software was freely downloaded from the Microsoft Store (https://apps.microsoft.com/detail/9nblggh33n0n). The distance between the pot and the receiver and the orientation of the receiver were kept the same for all measurements due to the very high sensitivity of the receiving sensor.

### Natural soil lab experiment

**Fig. 2:**
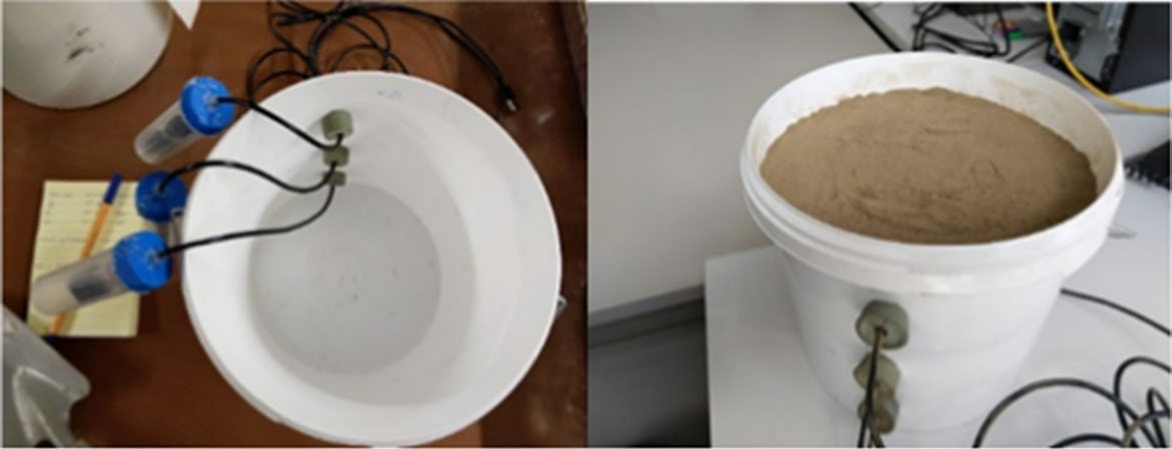
The experimental setup for the test with natural soil

In this experiment, natural soil material was used as a measurement medium. The materials and devices for this experiment are listed in Table 2. Oven-dried soil material was passed through a 2-mm sieve and packed into a plastic pot with a bulk density of 1.5 g/cm^3^. Three Wi-Fi signal transmitters were embedded in small sealed plastic tubes of 2 cm in diameter and 8 cm in length and positioned horizontally at various depths in the pot (Fig. 2). The vertical distance between each transmitter was 5 cm. The soil was saturated by applying water at the surface and allowed to evaporate, and the VWC was estimated daily using a balance. The RSSI was measured repeatedly once a day at the same time for all transmitters, using a laptop computer as the receiver and signal analysis device. The measurements were carried out for approximately 1 month under natural evaporation conditions. The distance and alignment between transmitter and receiver were kept the same all the time.

**Table 2:** Materials/devices used in natural soil experiment

Materials/devices	Specification/roles
JKI soil, packed	Loamy sand textured soil (disturbed) with 0.65, 0.29, and 0.06 kg/kg content of sand, silt, and clay respectively. The EC for the 1:1 soil solution extract was 0.59 dS/m
Three tp-link 5/2.4G dual-band Wi-Fi adapter	Installed at different depths inside the soil to be the source of the transmitted Wi-Fi signal
USB cables	Three × 1.8 m extension cables to connect the adapters to the desktop computer
Desktop computer	Connect to the Wi-Fi adapters through USB cables to control the on/off Wi-Fi signal
Laptop computer	Used as Wi-Fi receiver to measure the signal strength using “Wi-Fi analyzer” software
Sample containment	A plastic pot with a cylindrical shape, measuring 250 mm in diameter and 270 mm in height, was used to contain the sample

### Influence of EC on the relationship between RSSI and moisture content

To investigate the influence of EC on the relationship between RSSI and volumetric water content, we performed experiments that have the same setup as the first experiment with quartz sand. However, the used cables and Wi-Fi adapters were different. The transmitter was installed in the center of a quartz sand sample. Table 3 shows the materials that are used.

**Table 3:** Materials/devices used in the salinity experiment

Materials/devices	Specification/roles
Quartz sand	Pure white quartz sand, 0.1–0.3 mm particle diameter, no organic content
Realtek 2.4G USB Wi-Fi adapter	Installed inside the sand to be the source of the transmitted signal. [Conrad electronics, < 10 €]. Used with (0, 2.5, and 4.8 dS/m) EC levels
BIGtec 2.4G USB Wi-Fi Adapter (1)	Installed inside the sand to be the source of the transmitted signal. Used with (4.8 (2) dS/m) EC level
BIGtec 2.4G USB Wi-Fi Adapter (2)	Installed inside the sand to be the source of the transmitted signal. Used with (8.9 dS/m) EC level
USB cable	1.8 m USB extension cable to connect the adapter to the desktop computer
Desktop computer	Connected to the Wi-Fi adapter through a USB cable to control the on/off switch of the Wi-Fi signal
Laptop computer	Used as Wi-Fi receiver to measure the signal strength using “Wi-Fi analyzer software”
Sample containment	A plastic pot with a cylindrical shape, measuring 120 mm in diameter and 120 mm in height, was used to contain the sample
Fluid	Distilled water, NaCl solutions with electrical conductivities of 2.5, 4.8, and 8.9 dS/m at 20 °C

The quartz sand was washed with distilled water, oven-dried, and packed to 1.6 g/cm^3^ dry bulk density. The RSSI was measured for the dry samples to determine the initial signal before starting the experiment. After that, the sample was saturated from the surface using distilled water and left to natural evaporation. The changes in RSSI against the changes in VWC were daily measured. When the sample got dry, the same procedure was repeated two times using different concentrations of NaCl solutions (2.5 and 4.8 dS/m) for saturation. Unfortunately, we lost the transmitter due to a water leakage problem in the middle of the 4.8 dS/m experiment. Therefore, the experiment for the 4.8 dS/m was repeated using a different transmitter as well as a different transmitter for the 8.9 dS/m. In summary, the experiments involved different devices and solutions with electrical conductivities of 0, 2.5, 4.8, and 8.9 dS/m at 20 °C (0, 1280, 2560, and 5120 mg/L). In order to isolate the influence of using different devices (different values for initial RSSI) in the experiments, the relationships between signal attenuation (signal path loss, *SPL*) and VWC were inspected. The attenuation is calculated as follows:

7 $$SPL(dBm)=RSSI_o-{RSSI}_x$$

where $RSSI_o$ is the initial signal strength for the experimental setup of the treatment before saturation (dry sample) and ${RSSI}_{x}$ is the value of $RSSI$ at the $(x)$ value of VWC.

The changes in mean bulk EC for the samples were calculated according to the changes in soil water content. We have not explicitly taken into account the internal uneven distribution of salt concentration caused by evaporation.

### Signal transmission within a natural field soil

**Fig. 3:**
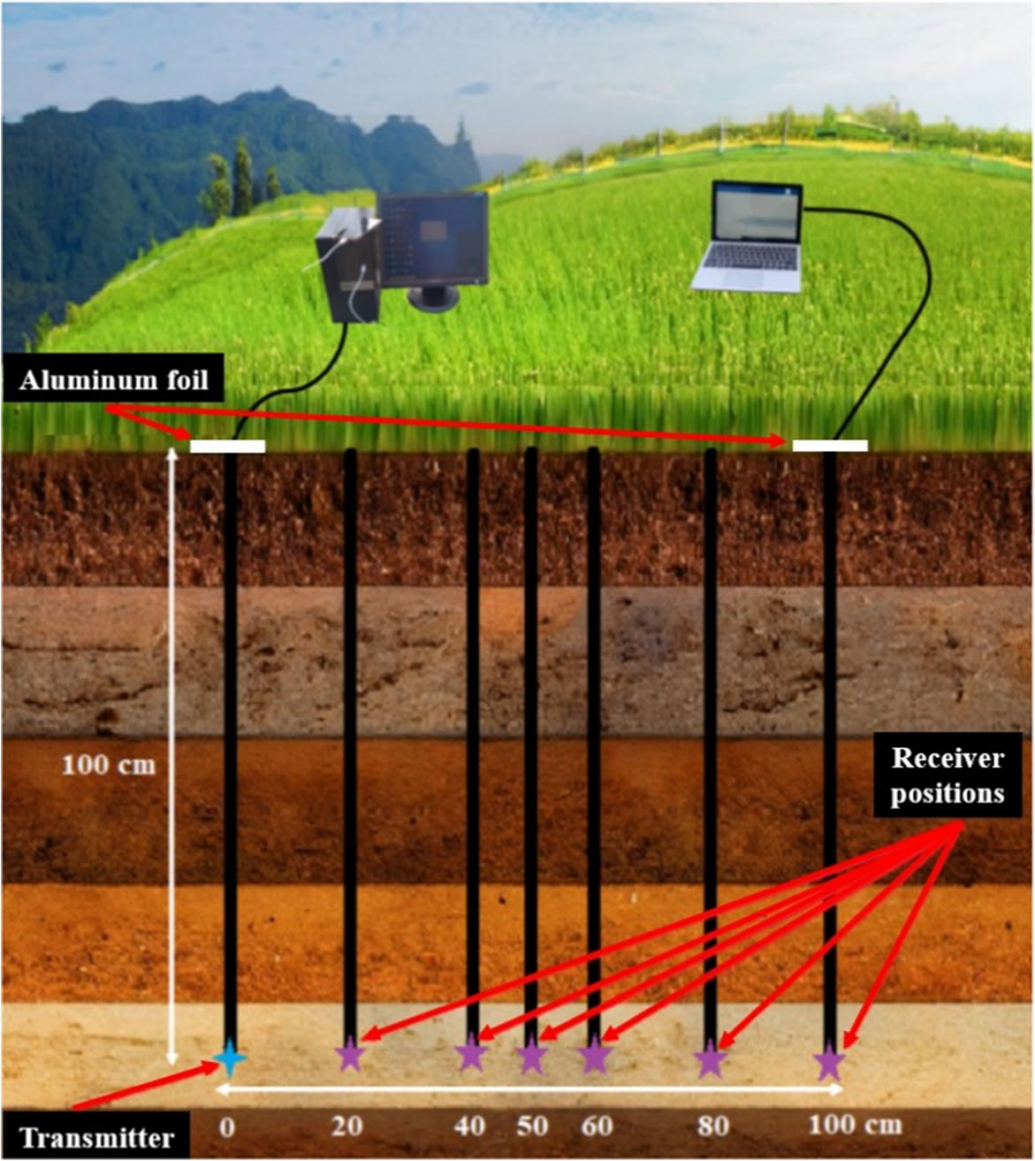
Illustration of signal transmission test in soil under natural field conditions

This field experiment aimed to address two primary questions: how is the distance the signal could traverse through natural soil, and how is the relationship between signal attenuation and the length of the signal path in the soil. The field trials were conducted at the Julius Kühn Institute (JKI), Messeweg, Braunschweig. Six boreholes, each with a diameter of 5 cm, were drilled into the soil at horizontal distances of 20, 40, 50, 60, 80, and 100 cm from the transmitter borehole, all reaching a depth of 100 cm. A representative soil sample was collected from 100 cm depth to determine the gravimetric water content (GWC). The transmitter was then inserted into the soil hole at a depth of 100 cm, after which the signal strength was measured at the same depth in the other holes, which were positioned at varying distances from the transmitter hole. The experimental setup is illustrated in Fig. 3. Details regarding the materials and equipment used can be found in Table 4.

**Table 4:** Materials/devices used in signal transmission experiment

Materials/devices	Specification/roles
Soil	Light textured JKI field soil (sand), GWC 17%
Mobile phone	Used as a source to connect the desktop computer to the Internet
USB type C cable	Used to connect the computer to the Internet through mobile phone
Aluminum foil	Two small sheets used to cover the holes during measurements
tp-link antenna 2.4/5G dual-band Wi-Fi adapter	Inserted to the soil auger hole and used as a transmitter (connected to the desktop computer)
logilink 2.4/5G dual-band Wi-Fi adapter	Inserted to auger holes and used as receiver (connected to the laptop computer)
USB cables	Two × 1.8 m extension cables to connect the adapters to the desktop computer
Desktop computer	Connect to the Wi-Fi transmitter through a USB cables to control the on/off Wi-Fi signal
Laptop computer	Connected to the Wi-Fi receiver to measure the signal strength using “Wi-Fi analyzer” software

The experimental setup was as follows: data was shared from the mobile phone to the computer, the computer activated the signal via a transmitter located 100 cm below the soil surface, the signal was received by a receiver located at the same depth but at different distances from the transmitter, and finally, the RSSI was measured via a laptop connected to the receiver.

To ensure that no signal from the transmitter reached the laptop through the air-only phase (i.e., via the open boreholes), which could lead to measurement errors, we checked the Wi-Fi signal before connecting the receiver to the laptop. If the target signal (the signal from our transmitter) was not detected in the air, we then connected the receiver and measured the received signal as the signal transmitted through the ground. Additionally, we covered the openings of the auger holes with aluminum foil during measurements.

In the same experiment, signal attenuation was measured in the air at three distinct distances: 0, 50, and 100 cm. The measurement at 0 cm was taken with the receiver in direct contact with the transmitter.

### Integrated soil moisture sensing experiment in the field

This final experiment was conducted at the same location in the JKI field. The aim was to demonstrate the relationship between Wi-Fi signal strength and moisture content under natural field conditions. Materials and devices for this experiment are listed in Table 5.

**Table 5:** Materials/devices used in soil moisture field experiment

Materials/devices	Specification
Field soil	Light textured soil (sandy)
Mobile phone	Used as a source to connect the desktop computer to the Internet
USB type C cable	Used to connect the computer to the Internet through mobile phone
AC1300 antenna 2.4/5G dual-band Wi-Fi adapter	Three antennas installed (horizontally) in the soil at different depths and used as transmitters
USB cables	Three × 3 m extension cable to connect the transmitters to the desktop computer
Desktop computer	Connect to the Wi-Fi transmitters through USB cables to control the on/off Wi-Fi signal
Laptop computer	Used as the Wi-Fi receiver to measure the signal strength using “Wi-Fi analyzer” software

**Fig. 4:**
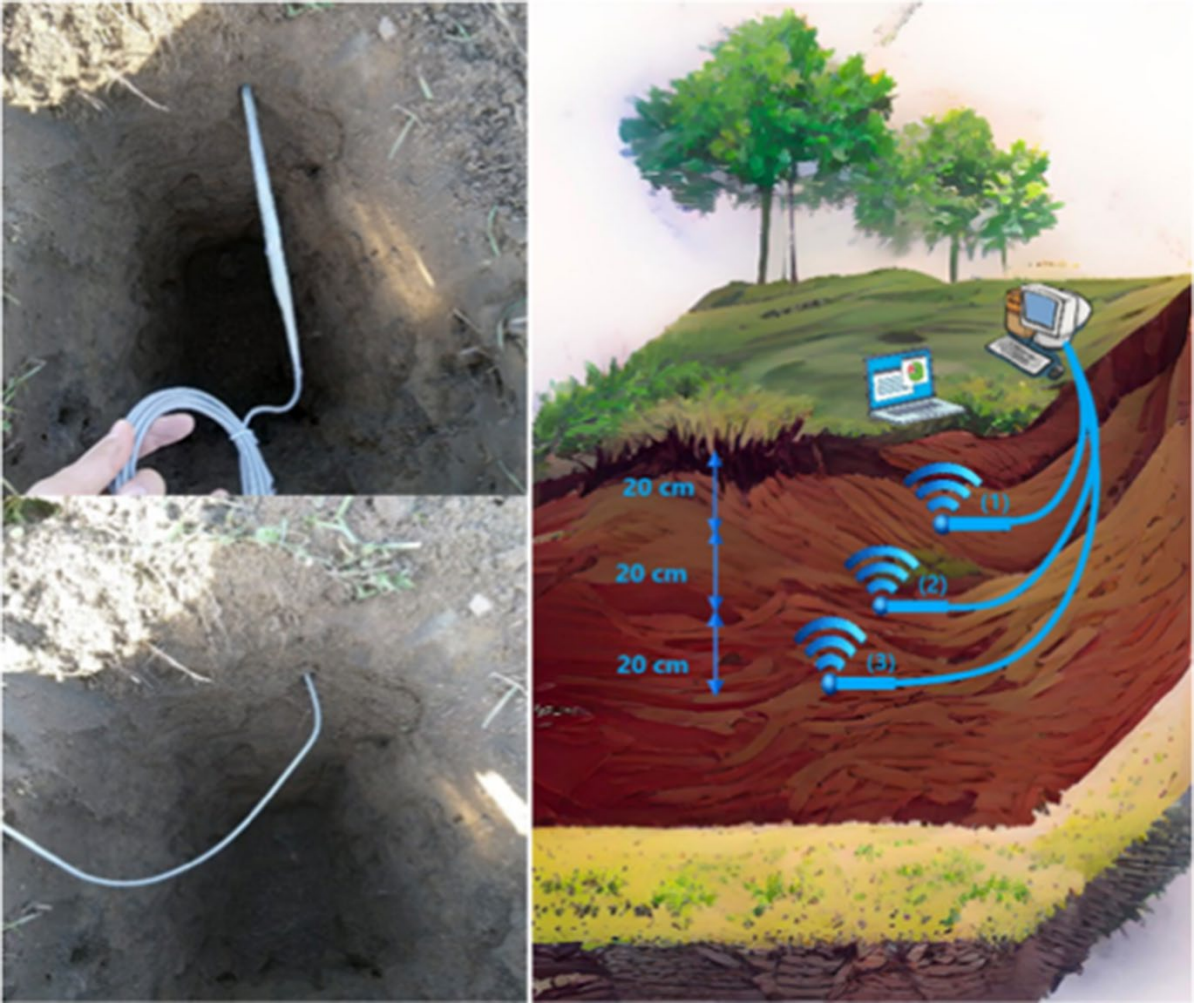
Experimental setup for the test with natural soil

In this field experiment, three dual-band Wi-Fi antennas were installed horizontally at three different depths below the soil surface (20, 40, and 60 cm). RSSI was measured at the soil surface directly above the transmitters using a laptop computer. The antennas were wrapped using waterproof tape prior to the installation to prevent water from entering the interior of the transmitters, which could cause damage. Measurements were made by turning on the signal from the desktop computer for each transmitter individually and measuring the RSSI with the laptop at the ground surface. Soil samples were additionally collected from representative depths and at the same measurement times to determine GWC as reference measurements in the laboratory. The images in Fig. 4 show the installation and measurements in this experiment.

### Data analysis

The Microsoft Excel tool was used for data visualization and to generate scatter plots illustrating the relationships between RSSI and soil VWC. Curve fitting was performed to analyze these relationships. The coefficient of determination (*R*^2^) values were calculated for the fitted curves. Error bars were included in the scatter plots to represent the variability or uncertainty associated with the measurements. Errors in RSSI are given by the limited resolution of 1 dBm; errors in water contents from weighing are roughly estimated to be ± 2% GWC.

## Results

### Quartz sand experiment

**Fig. 5:**
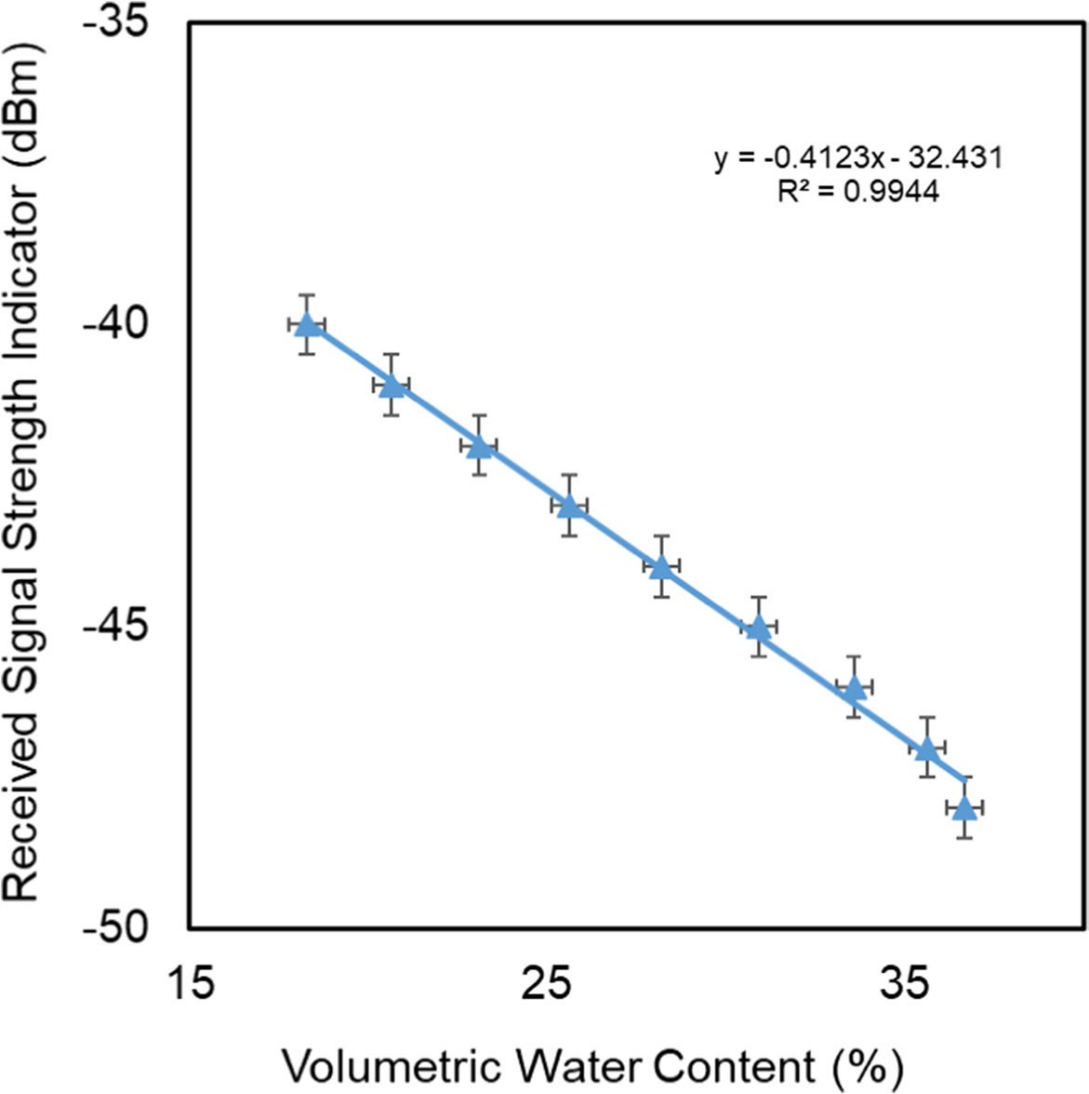
Measured received signal strength indicator (RSSI) vs. mean volumetric water content in the pot. Error bars indicate the standard deviation of VWC and resolution of RSSI

The changes in signal strength, in response to the changes in volumetric water content for the quartz sand experiment, are presented in Fig. 5.

Results of the evaporation experiment show that the decrease in mean water content within the sample correlates closely with an increase in signal strength. The relationship between RSSI and VWC is linear, with a slight exception at the highest water content. The exception might be related to nonlinearity in the water content profile in the pot close to water saturation (Peters & Durner, 2006). The relationship was very strong, with a high value for the coefficient of determination (*R*^2^ > 0.99). It is worth mentioning here that, due to the lockdown during the pandemic, the measurements were halted before the sample reached a fully dry condition, as we were unable to access the lab.

### Natural soil experiment

**Fig. 6:**
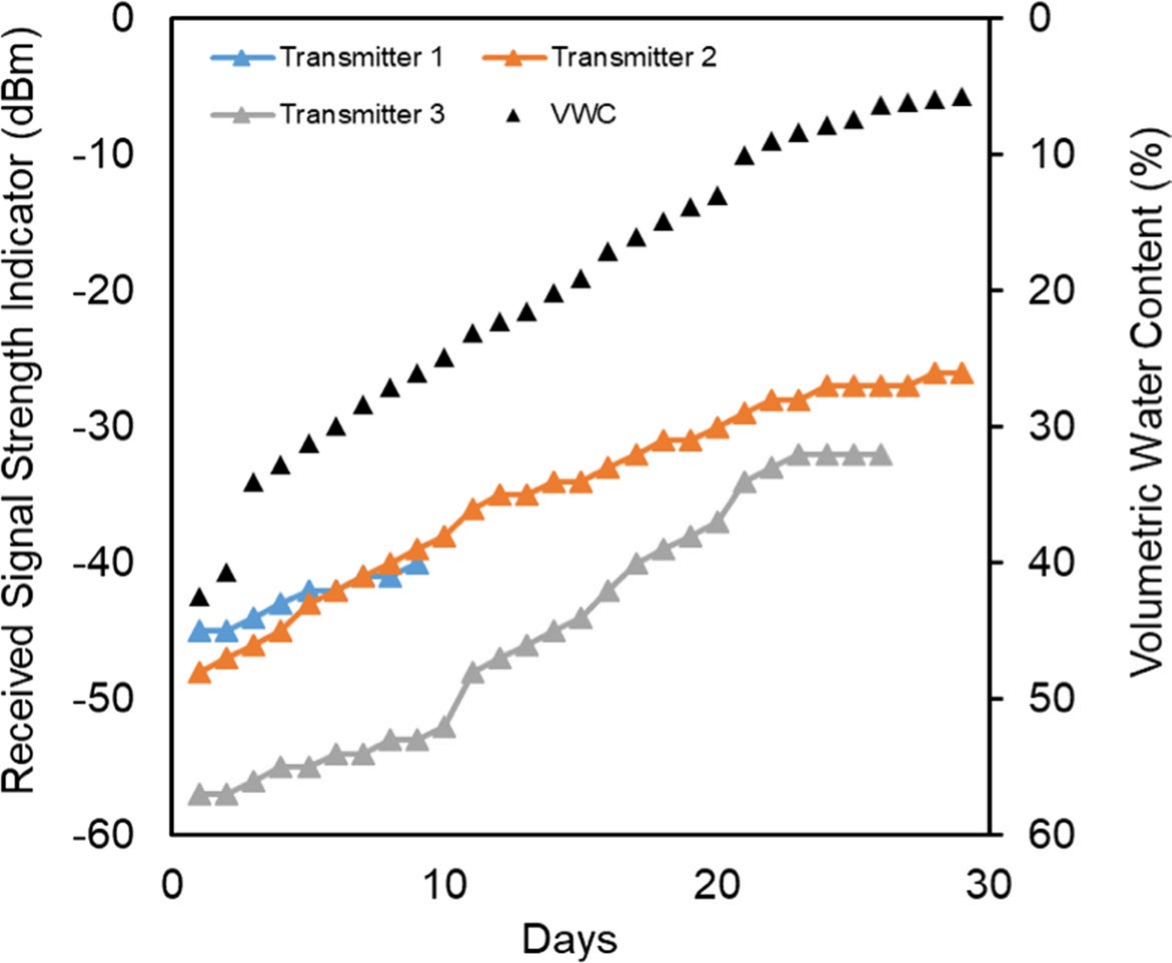
Development over time of the VWC and the signal strength of the three Wi-Fi transmitters in natural soil

**Fig. 7:**
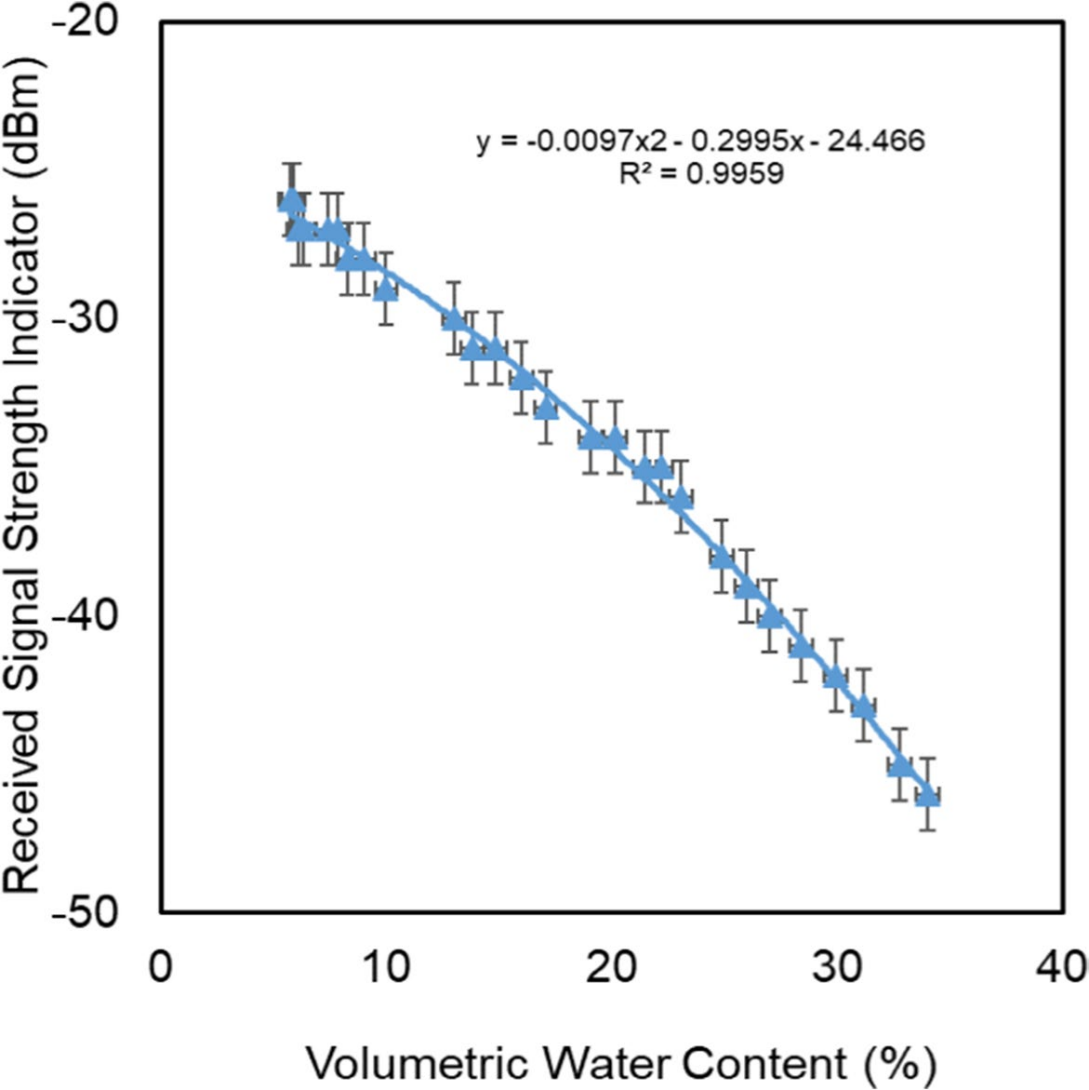
Relationship between VWC and Wi-Fi signal strength (RSSI) in JKI soil

Figure 6 shows the temporal evolution of the daily RSSI data and the corresponding VWC for the pot evaporation experiment with natural soil. The value of RSSI follows the changes in soil water content, with a notable rapid change in VWC between days 2 and 3. This rapid change is likely due to above-average temperatures during this period, which accelerated the rate of water evaporation. Unfortunately, we lost transmitter one (upper transmitter) after 9 days and transmitter three (lower transmitter) after 26 days. However, the results clearly show again a close relationship between soil volumetric water content and RSSI. This is illustrated in Fig. 7, where RSSI for transmitter number two (installed in the center of the sample) is plotted as a function of mean VWC.

The relationship between the RSSI and VWC is again close (*R*^2^ > 0.99), but slightly nonlinear, which is different from the first experiment. This difference may be due to the use of soil material, which can differ significantly from the quartz sand used in the first experiment. However, both experiments conducted under controlled laboratory conditions may also differ from real field conditions. Improving measurement resolution, like using better software or a signal analyzer, could greatly boost measurement accuracy, as much of the error is due to the limited 1 dBm resolution of the current Wi-Fi analyzer.

### Influence of EC on the relationship between RSSI and moisture content

**Fig. 8:**
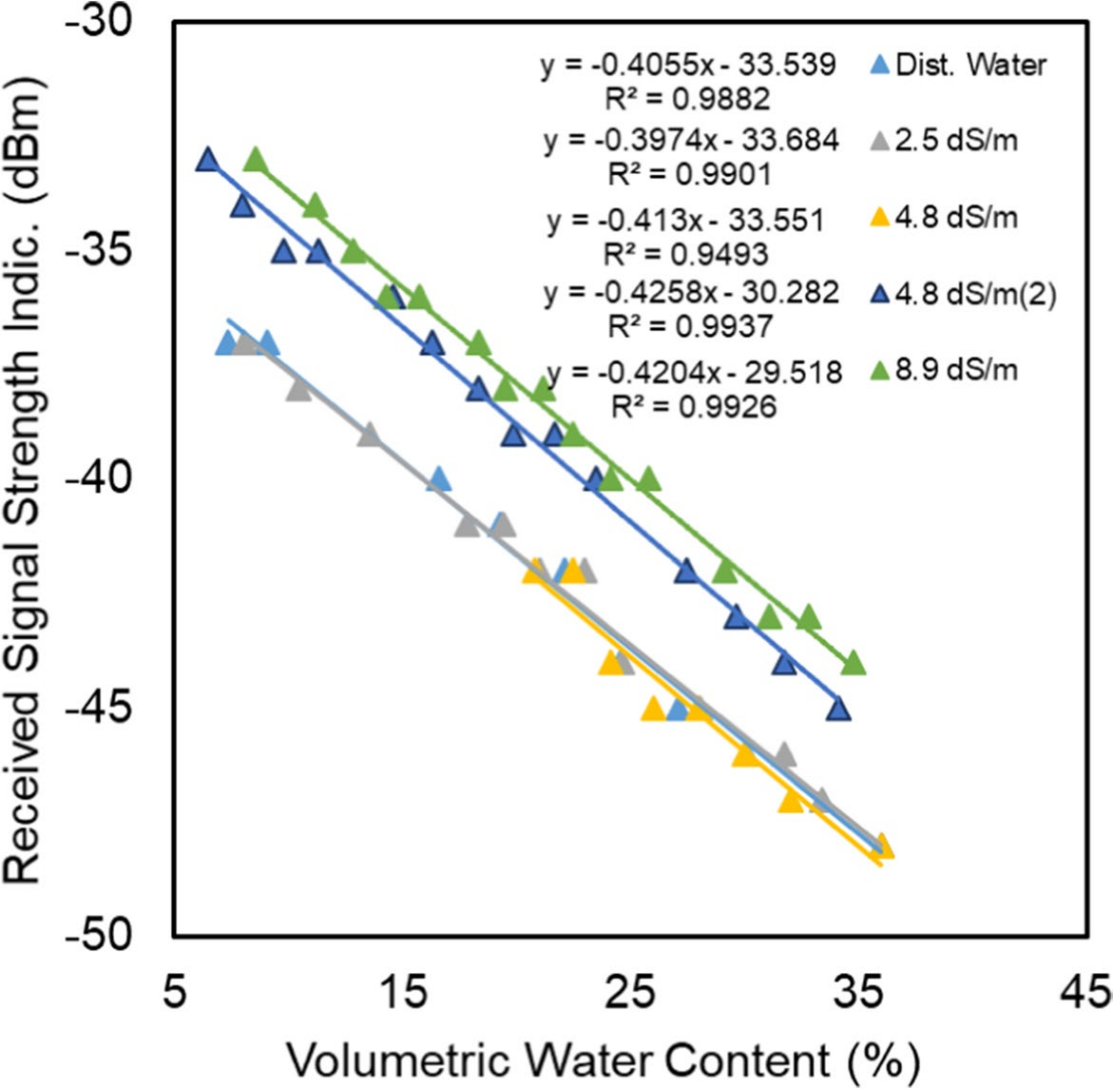
RSSI vs. VWC in quartz sand at different EC levels

Figure 8 shows the relationships between RSSI and VWC at different levels of electrical conductivity for the solutions that are used to saturate the samples. For the levels of 0, 2.5, and 4.8 dS/m, measured with the same setup of transmitters (first run), the linear relationships were strong (*R*^2^ from 0.93 to 0.99), and both the slope and the offset of the relation were virtually the same. However, shifting in the linear relationships was observed for the treatments of 4.8 (second run) and 8.9 dS/m. The changes in the intercepts are due to the use of different transmitters in these runs.

We were forced to use different transmitters because the first transmitter was damaged at the 4.8 dS/m run. However, the slopes of the relationships were almost identical for the different transmitters. To subtract the influence of using different transmitters in the experiments, the signal attenuation was calculated (Eq. 7) and related to the VWC for all treatments.

**Fig. 9:**
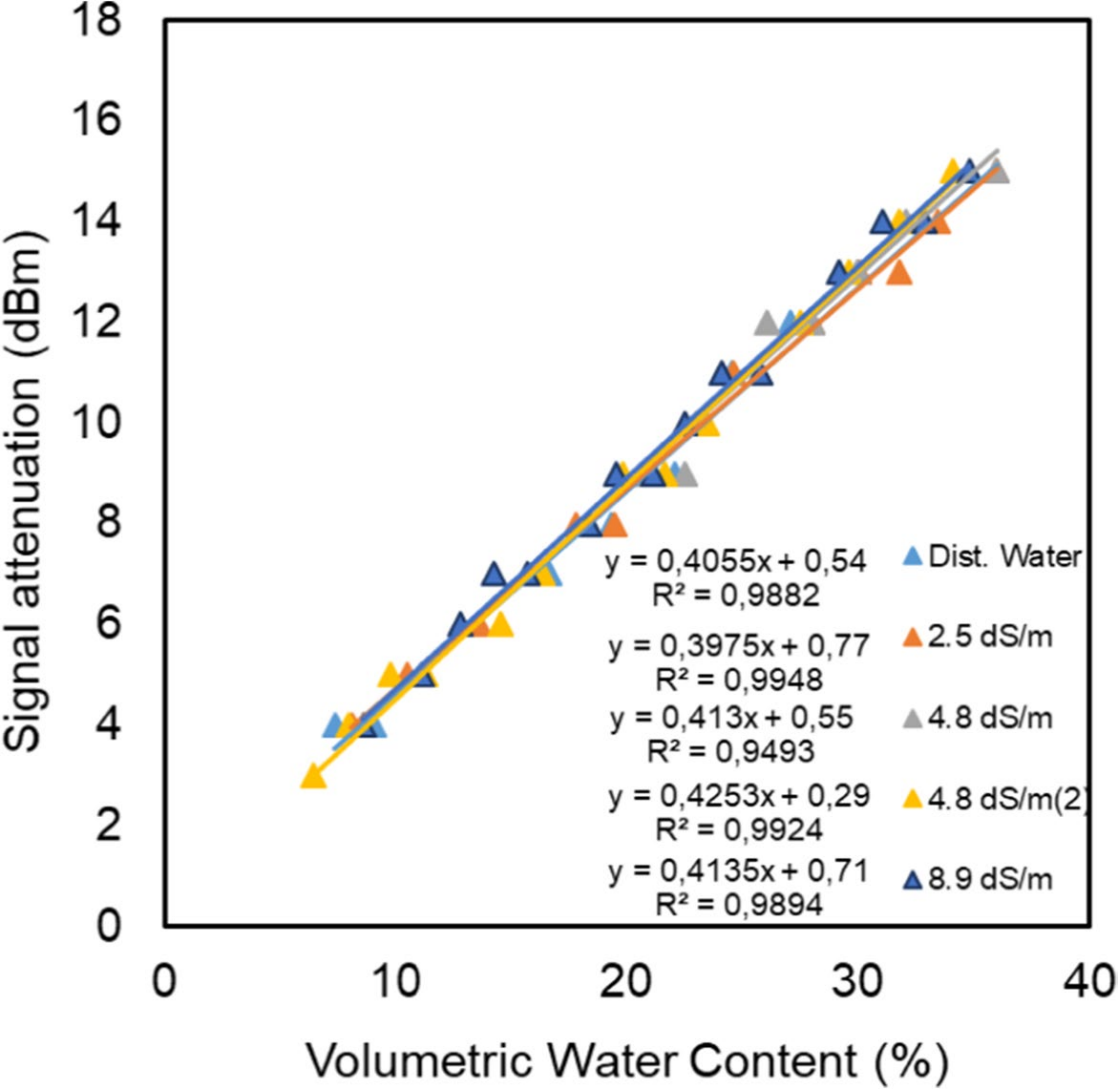
Signal attenuation vs. VWC in quartz sand at different EC levels

**Fig. 10:**
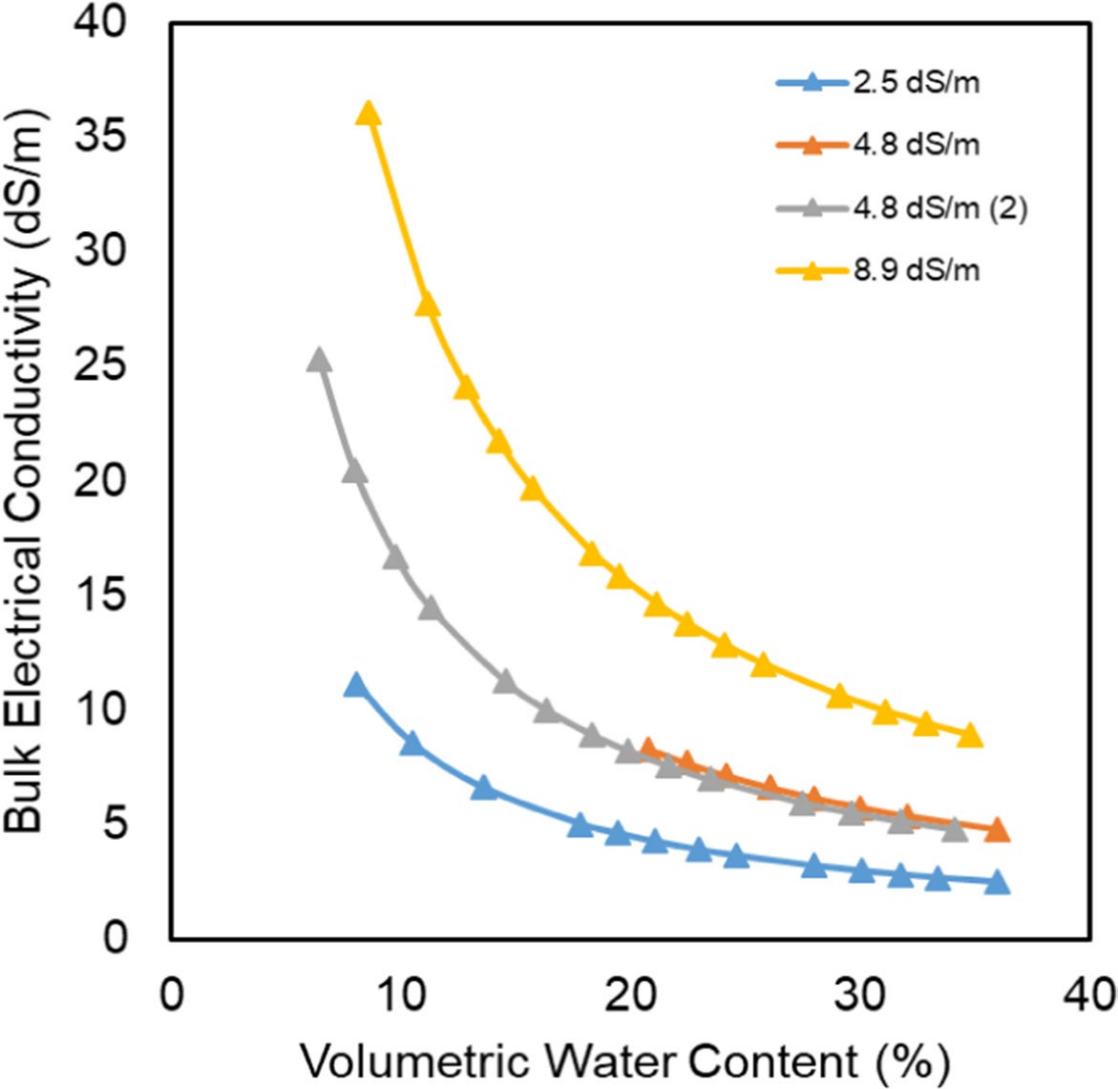
Changes in mean bulk EC in response to the changes in VWC

Figure 9 shows the relationships between signal attenuation and VWC at different levels of saline solutions that were used in saturation. The relationships are strong, with *R*^2^ values ranging between 0.94 and 0.99. Furthermore, there was no significant influence of the different levels of electrical conductivity on the signal attenuation, despite high levels of the mean bulk electrical conductivities (Fig. 10) that occurred during the experiments due to the evaporation that lead to the concentration of the saturation solutions.

Increasing the conductivity of the medium leads to an increase in the signal reflectance, causing a slowing down of the signal speed and an increase in the signal attenuation (Qureshi et al., 2016). However, the reason for the insignificant influence may be related to one or more of the following points:

1. It has been reported in the literature, e.g., Gardner et al. (2001), that the influences of EC on the measurements are negligible at frequencies greater than 50 MHz, and in our case, the operation frequency is 2400 MHz.
2. The evaporation process causes salts to be transported to the surface layer of the sample (Salman et al., 2023). Consequently, during the experiments, these salts do not intercept the flying path of the signal between the transmitter and receiver.
3. The used concentrations are lower than the thresholds of the significant influence of the EC in the measurements.

### Signal transmission in a natural soil in field

**Fig. 11:**
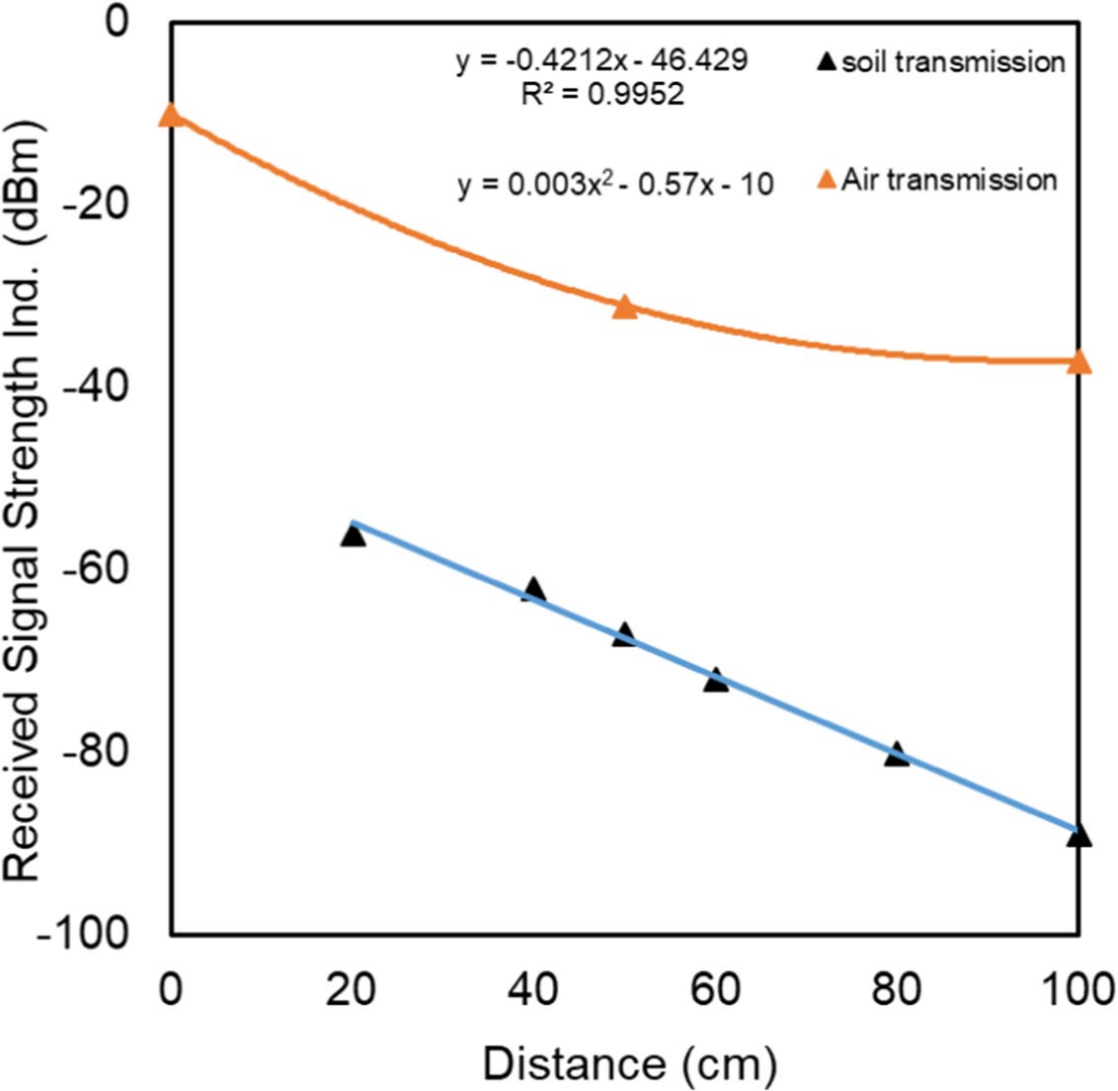
Results of the signal transmission experiment in the field

The results of the signal transmission in the field soil are shown in Fig. 11. The results indicate that at given soil conditions and with the measurement components used, the signal barely passed 1 m, when it reached the end of the measurable range for the used software (approximately − 90 dBm). Signal attenuation depended linearly (*R*^2^ > 0.99) on the length of the traveled path in soil.

The length of the signal travel path within the soil body is an important factor as it determines the flexibility in selecting the size of the representative soil sample based on the specific objectives of the soil moisture measurements. It is important to note that both the generated and transmitted signals, as well as the received signal strength, are dependent on the device and software used. In other words, the length of the measured travel path through the soil is influenced not only by soil conditions (factors that affect the dielectric properties of the soil) but also by the devices and software utilized.

### Soil moisture sensing experiment in the field

**Fig. 12:**
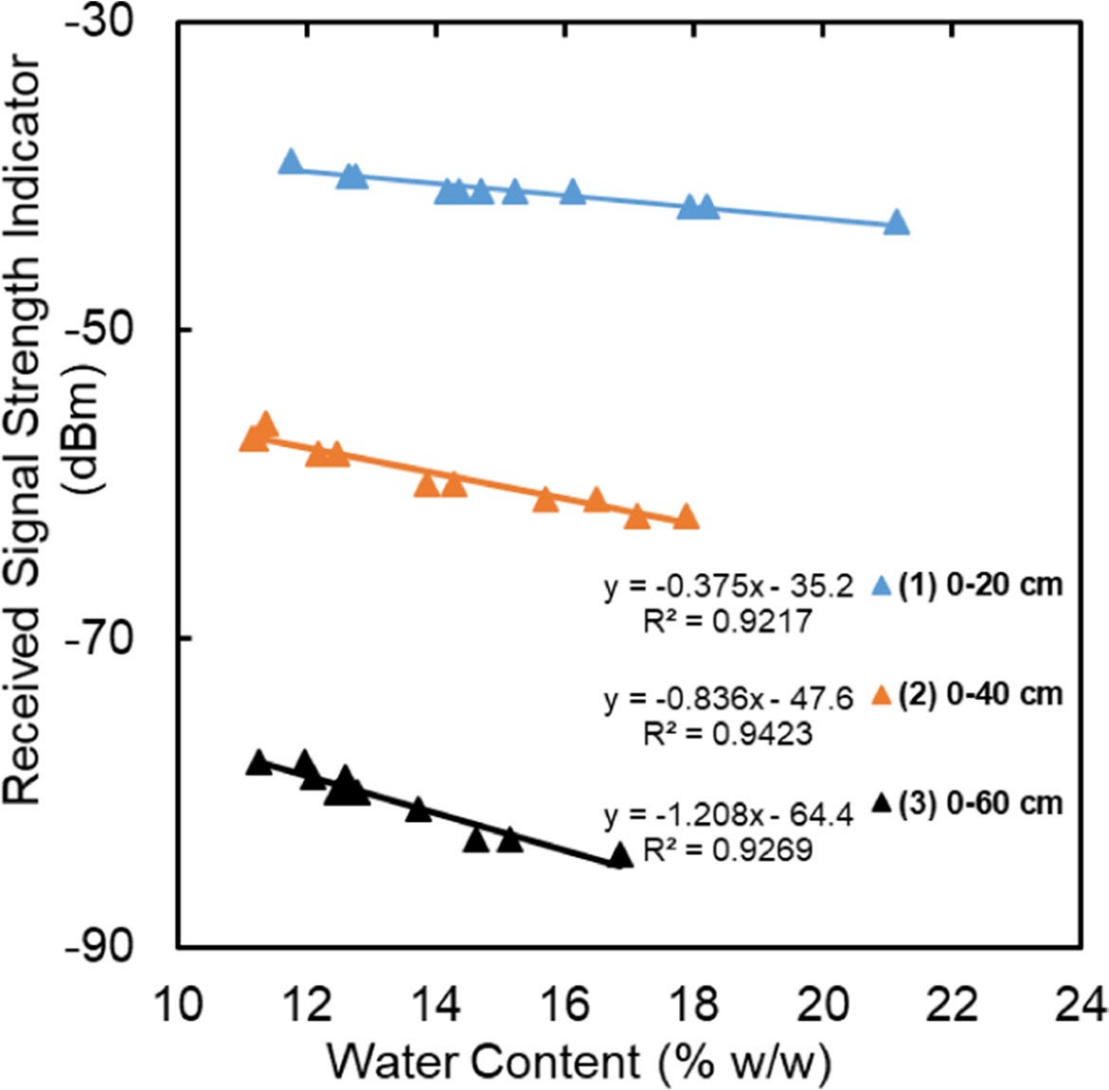
RSSI vs. integrated gravimetric water content in the field

The response of signal strength to the changes in GWC of the soil in the JKI field is presented in Fig. 12. The field experiment results indicate strong linear relationships between RSSI and GWC for all transmitters, with *R*^2^ values of 0.92, 0.94, and 0.93 for the upper, middle, and lower transmitters, respectively. The measured signal strength at the soil surface represents an integrated amount of soil moisture for the depth between transmitter and receiver at the soil surface. Thus, it comprises the measurement of soil moisture in very large soil volumes compared to most of the existing technologies. Measuring soil moisture in different soil layers is possible using multi-transmitter/receiver systems and even reduces the problem of soil surface mirroring at greater depths.

## Discussion

We could show that the Wi-Fi signal transmission strength is sensitive to soil water content in a given setup and for a given soil. The relationship between RSSI and VWC is systematic and highly significant. A lot of work is still required to study and control many of the factors influencing the measurements:

1. Factors related to the creation of the Wi-Fi signal by the hardware (currently a desktop PC), particularly the constancy and stability of the signal.

2. Factors related to the emission of the transmitted signal by the adapter, namely adapter type, alignment and positioning, cable type, cable length, and cable orientation.

3. Factors related to the reception of the signal, namely the position and orientation of the receiver (currently a laptop).

4. Effect of signal analyzing software and/or signal analyzing devices.

Factors 1 to 4 are related to the reliability of the measurement setup and hardware/software configuration as such, not to the specific soil. If we can show that a reliable measurement (without specific calibration) is possible for a certain material, then the next points have to be investigated:

5. Effect of soil types and properties (particularly bulk density, gravel content, organic matter, clay content, parent material, and salinity).

6. Surrounding environmental conditions (e.g., temperature, air humidity, but also the role of external Wi-Fi signals, which are more or less ubiquitous in certain environments).

We are aware that the idea to establish water content measurements based on a single and very easily measurable signal variable may seem bold at first glance in view of the fact that, in addition to instrumental design factors (Factors 1 to 4), soil conditions such as salinity, bulk density, clay content, and organic matter also determine the attenuation of Wi-Fi signals in the soil. In order to contextualize these factors, however, it is important to recognize that they also cause problems for established measurement techniques for determining the electrical permittivity and often lead to the recommendation to calibrate the capacitive sensors or sensors working with waveguides in the field (Salman et al., 2021; Nieberding et al., 2023; Adle et al., 2024).

The measurement technique tested here for the first time not only is based on very cheaply available components but also reduces the major problem of close contact between sensor and soil, which is a most critical problem for sensors using waveguides (Stevenson et al., 2021). Provided the influences of the measuring device configuration are fixed using a standardized instrument, it appears to have a great potential, in particular, if the aim is the detection of changes in water contents rather than absolute values. It should be noted that, in this initial phase of concept validation, the current setup relies on both a desktop computer and a laptop, which increases costs and requires human interaction. We acknowledge that water leakage damaged some transmitters, requiring experiment repetition in the presented work. To address this, we plan to use more robust waterproofing materials in future work to ensure protection and maintain consistency across all devices. Moving forward, our next steps involve developing fully automated solutions, such as integrating microcontroller units, to enable remote and continuous data collection while keeping costs low.

## Conclusion and outlook

The use of Wi-Fi signal for soil moisture measurement appears to be promising towards developing a novel, cheap, easy, and relatively accurate solution for monitoring soil moisture. A particular advantage is that the sensing is across soil volumes, which means that it is not affected by the properties of the soil in the direct vicinity of electric waveguides. Additionally, the sample size measured is adaptable based on the distance between the transmitter and receiver, which can range from a few centimeters up to 1 m or even more with additional setups such as amplifiers.

The results of our preliminary studies indicate that Wi-Fi signals can travel distances in moist soil in the range of a meter or more which makes it for relevant for a wide range of applications in soil science, hydrology, and engineering. Furthermore, increasing the measurement depth can be achieved by employing multi-transmitter/receiver systems that alternate between transmitter and receiver roles to monitor soil moisture at different layers and along longer paths within soil horizons.

Such approach within the soil body also assists in mitigating the measurement errors caused by the soil surface mirroring effect. This effect arises when the signal travels from the air phase above the soil surface to the receiver positioned beneath the soil surface (as suggested by methodologies outlined in Ding & Chandra, 2019a, 2019b), which may encounter challenges associated with soil surface roughness and the presence of a water layer above the soil surface resulting from rainfall or irrigation practices. The use of multi-transmitter/receiver below the soil surface provides a more effective solution to address these issues.

For the next steps, we are looking to establish an independent device for soil moisture sensing according to the mentioned Wi-Fi basics. Furthermore, we are thinking of different designs for the sensing system to be useful for different field and lab purposes. However, extensive experimental studies are still required to investigate the accuracy and limitations of employing signal properties (e.g., time of fly, attenuation, phase shifting) on soil moisture sensing. Extensive studies in a wide range of soils and environmental conditions are also required to ensure robustness and reliability.

## Data Availability

No datasets were generated or analysed during the current study.
